# Association of Polymorphic Variants of miRNA Processing Genes with Larynx Cancer Risk in a Polish Population

**DOI:** 10.1155/2015/298378

**Published:** 2015-11-25

**Authors:** Ewa Osuch-Wojcikiewicz, Antoni Bruzgielewicz, Kazimierz Niemczyk, Olga Sieniawska-Buccella, Alicja Nowak, Anna Walczak, Ireneusz Majsterek

**Affiliations:** ^1^Department of Otolaryngology, Medical University of Warsaw, Banacha 1a Street, 02-097 Warsaw, Poland; ^2^Department of Clinical Chemistry and Biochemistry, Medical University of Lodz, Hallera 1 Square, 90-647 Lodz, Poland

## Abstract

Laryngeal cancer (LC) is one of the most prevalent types of head and neck cancer. An increasing interest has been focused on the role of microRNA (miRNAs) in LC development. The study group consisted of 135 larynx cancer patients and 170 cancer-free individuals. Nine polymorphisms of pre-miRNA processing genes,* DROSHA* (rs6877842),* DGCR8* (rs3757, rs417309, and rs1640299),* RAN* (rs14035),* XPO5* (rs11077),* DICER1* (rs13078 and rs3742330) and* TARBP2* (rs784567), were performed by TaqMan SNP Genotyping Assay. It was found that the frequency of the GT and the TT polymorphic variants of* XPO5* gene were higher in LC patients than in controls (*p* < 0.0001 and *p* = 0.000183, resp.). In turn, the frequency of the CT genotype of* RAN* gene was higher in controls than in LC patients (*p* < 0.0001). The TT and the AG of* DICER1* gene (*p* = 0.034697 for rs13078 and *p* = 0.0004 for rs3742330) as well as the AG and the GG genotypes of* TARBP2* gene (*p* = 0.008335 and *p* < 0.0001, resp.) were associated with higher risk of LC occurrence. Our data suggested that polymorphisms of miRNA processing genes might be useful as predictive factors for the LC development.

## 1. Introduction

Laryngeal cancer (LC) is one of the most prevalent types of head and neck cancer (HNC) in the contemporary world. Despite the improvement of surgery, chemotherapy, and radiotherapy, the 5-year survival rates are less than 60% [1]. In the last years, increasing interest has been focused on the role of microRNA (miRNA) in cancer development and progression. As in other malignancies, miRNA regulates several oncogenes and tumor suppressors, driving the growth, proliferation, metastatic attitude, and drug resistance [2–4].

Among many environmental (like tobacco smoking, alcohol consumption, or Human Papilloma Virus infection) and genetic factors that can cause HNC, it is still unknown whether factors engaged in microRNA (miRNA) processing can be one among the factors that can put an individual at risk of the disease [5, 6]. miRNAs are a class of endogenous, short (approximately 22 nt) noncoding RNAs that have emerged as major regulators of posttranscriptional gene expression. Thus, they can decrease the level of many proteins, despite the high level of transcription. Elements that are crucial for proper working of miRNA are proteins involved in miRNAs processing, because they reorganize the structure of premature pre-miRNAs into fully functioning miRNAs [7].

miRNA genes are first transcribed by RNA II polymerase into primary miRNAs (pri-miRNAs) with several hundred nucleotides. Processing of primary miRNAs (pri-miRNA) by the nuclear RNase III DROSHA within the microprocessor complex also including DGCR8 (DiGeorge syndrome critical region gene 8) produces the 70 to 100 nt pre-miRNAs. The pre-miRNAs are then exported into the cytoplasm by the Exportin-5/Ran-GTP complex and cleaved by DICER as a part of the RNA-induced silencing complex's loading complex including TARBP2 and AGO2. This complex also includes GEMIN3 and GEMIN4 and contributes to both miRNA processing and target gene silencing. miRNAs regulate gene expression in animals and plants through binding to the 3′ untranslated region (UTR) of the mRNAs of their target genes and leading to mRNA cleavage or translation repression [8–10]. Thomson et al. [11] have shown that the repression of mature miRNAs is not consistent with the reductions in the primary miRNA transcripts, suggesting the existence of altered regulations of miRNA processing in human cancers. Aberrant expression of miRNAs contributes to the etiology of many common human diseases, especially cancers [12, 13].

Some changes, like single nucleotide polymorphisms (SNPs), in the structure of genes encoding miR processing proteins may affect their structure or expression level. Although SNPs have been widely implicated in HNC development [14], such evidence is lacking for miRNA biogenesis pathway genes. Thus, the aim of our study is to evaluate the connection of prevalence of LC with SNPs occurring in the following genes:* DROSHA* (rs6877842),* DGCR8* (rs3757, rs417309, and rs1640299),* RAN* (rs14035),* XPO5* (rs11077),* DICER1* (rs13078 and rs3742330), and* TARBP2* (rs784567). The new data may bring a new knowledge of genetic factors that may affect the risk of LC development. This may be useful as a screening factor that may help to classify the individuals to the group of higher risk and enroll them for the special prophylactic program.

## 2. Materials and Methods

### 2.1. Subjects

All subjects included in the study were unrelated white people living in Poland. The study group consisted of 135 patients (118 men and 17 women, mean age: 62 ± 9) with diagnosed head and neck cancer localized in larynx. Control group consisted of 170 cancer-free individuals (149 men and 21 women, mean age: 67 ± 14). Cancer type was confirmed with histopathology examination.

The study cohort included consecutive patients who underwent total or partial laryngectomy operation at Public Central Clinical Hospital in Warsaw, Poland. Macroscopic tumor samples were collected as far as possible from the necrosis area. As controls, noncancerous laryngeal mucosa tissue specimens were obtained from people who had had a total or partial laryngectomy for a benign condition. Tissues' samples obtained during surgery were immediately fixed in 10% buffered formalin for at least 4 h and processed for paraffin embedding.

Prior to examination, the patients and control subjects did not receive medicaments like antibiotics or steroids. Patients enrolled for the examination were analyzed according to cancer staging system of the TNM Classification of Malignant Tumors that describes the extent of cancer in a patient's body: T describes the size of the tumor and whether it has invaded nearby tissue, N describes metastasis to regional lymph nodes, and M indicates the presence of distant metastasis (spread of cancer to another organs).

Using the TNM system the cancer burden can be grouped into following stages: Stage I, T1, N0, M0; Stage II, T2, N0, M0; Stage III, T3, N0, M0 or T1–3, N1, M0; Stage IVA, T4, N0-1, M0 or any T, N2, M0; Stage IVB, any T, N3, M0; and Stage IVC, any T, any N, M1. According to TNM staging, our study included 10 cases of Stage I, 8 cases of Stage II, 55 cases of Stage III, 56 cases of Stage IVA, and 5 cases of Stage IVB. In evaluated patients group there were no cases of distant metastases.

Additionally, the neoplastic grading was also applied: G1, well differentiated (low grade) tumor; G2, moderately differentiated (intermediate grade) tumor; G3, poorly differentiated (high grade) tumor; and G4, undifferentiated (high grade) tumor. Within the patients group subjects were classified as smokers for at least 10 years, 10 to 40 years, and more than 40 years. The smoking attitude of head and neck cancer group was also analyzed for nonsmoking patients, patients smoking up to 20 cigarettes per day, and patients smoking more than 20 cigarettes per day. All patients and controls subjects were recruited from medical units of Head and Neck Neoplasm Surgery Departments, Medical University of Warsaw, Poland. The study was approved by the Ethics Committee of Medical University of Warsaw and written consent was obtained from each patient or healthy subject before enrolling in the study.

### 2.2. DNA Isolation

DNA was isolated from the formalin-fixed, paraffin-embedded tissues using the BiOstic FFPE Tissue DNA Isolation Kit (MO BIO), according to manufacturer's instructions. 10 mg of paraffin-embedded tissue was used for preparation. Samples were heated at 55°C in an optimized wax melting buffer and Proteinase K to completely digest the tissue. Then, a 90°C heating step was used to remove cross-links in the DNA and ensure successful PCR. The samples were then mixed with a chaotropic salt binding buffer and 100% ethanol for binding to silica filters. Impurities were washed from the column and pure DNA was eluted in a low salt buffer (10 mM Tris, pH 8.0). The purity and amount of DNA were evaluated using Synergy HT microplate reader (BioTek). After extraction, the DNA concentration was measured photometrically and the DNA was diluted to a concentration of 5 ng/*μ*L.

### 2.3. Genotyping Assay

**Figure 1:**
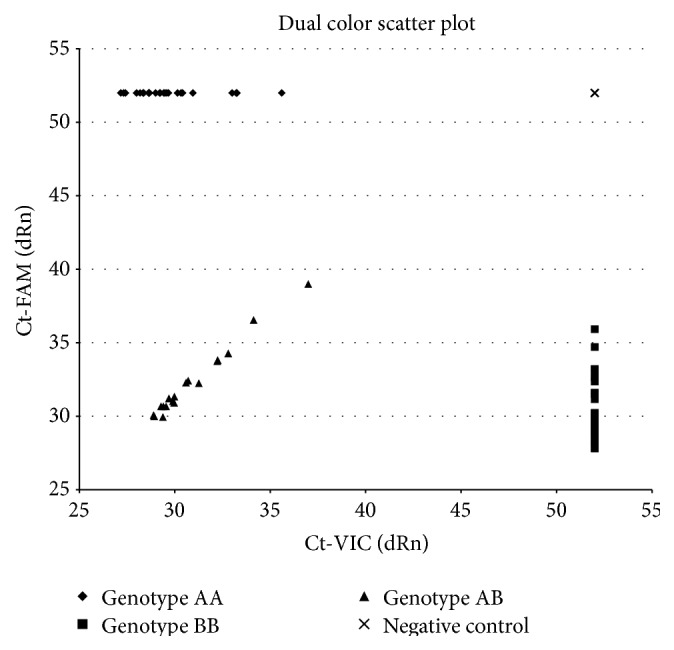
Representative allelic discrimination plot of genotyping using TaqMan SNP Genotyping Assay. Diamonds: homozygous AA; squares: homozygous BB; triangle: heterozygous AB. Cross sign represents no template negative controls.

The aim of this research was to obtain the association of polymorphic variants of genes involved in miRNA processing with the head and neck occurrence risk. In this case-control study, we wanted to evaluate the effects of 9 selected potentially functional single nucleotide polymorphisms (SNPs) in pre-miRNA processing machinery. SNP evaluation was obtained by TaqMan SNP Genotyping Assay with a commercially available primer probe sets (Applied Biosystems, Foster City, CA, USA) and TaqMan Genotyping Master Mix (Applied Biosystems, Foster City, CA, USA) performed on Mx3005P (Agilent Technologies, Santa Clara, CA, USA) according to manufacturer's instructions. Assay IDs were as follows: rs6877842-C_1153852_10, rs3757-C_2539471_1_, rs417309-C_2539468_20, rs1640299-C_7543549_20, rs14035-C_11351340_10, rs11077-C_3109165_1_, rs13078-C_7504801_10, rs3742330-C_27475447_10, and rs784567-C_9576934_20. For TaqMan Genotyping Assay, 5 ng of DNA template was used per reaction well. The mix for every single reaction was prepared as follows: 10 *μ*L TaqMan Genotyping Master Mix (2x), 1 *μ*L TaqMan Genotyping Assay Mix (20x), 9 *μ*L DNase-free, RNase-free water. Then, 20 *μ*L of reaction mix was added to the DNA template and briefly centrifuged to spin down the contents and eliminate air bubbles from the solutions. The reaction thermal profile was as follows: 95°C for 10 min, followed by 50 cycles of 95°C for 15 s and 60°C for 1 min. Representative allelic discrimination plot of genotyping using TaqMan SNP Genotyping Assay is presented in Figure 1. *x*-axis represents the emission for the A allele-specific probe labeled with 2′-chloro-7′-phenyl-1,4-dichloro-6-carboxyfluorescein (VIC), and *y*-axis represents the relative fluorescent emission for the B allele-specific probe labeled with 6-carboxyfluorescein (FAM). The resulting cluster plot shows strong fluorescent signals for each allele and clear separation between the three clusters, easily discriminating the two homozygous (diamonds: homozygous AA; squares: homozygous BB) and one heterozygous genotypes (triangle: heterozygous AB). Cross sign represents no template negative controls.

Evaluation of 20% of randomly chosen samples was performed again with RT-PCR to confirm the previously obtained results of genotyping and the results were 100% concordant.

### 2.4. Statistical Analysis

Genotype frequencies for each polymorphism were evaluated using the Hardy-Weinberg equilibrium test. Allele frequencies and the prevalence of genotypes were determined for the study and control groups and compared by *χ* ^2^ test. If expected frequencies in 2 × 2 contingency table were smaller than 5, Fisher's exact probability test was performed. Significant probability values obtained were analyzed for multiple testing using Bonferroni correction (*p* value after Bonferroni correction [*p*_corr_]). Statistical significance was defined as *p* < 0.05. All analyses were performed using STATISTICA 6.0 software (StatSoft, Tulsa, OK, USA).

## 3. Results

### 3.1. Distributions of Investigated Genotypes in Polish Population

Genotype frequencies for each polymorphism were evaluated using the Hardy-Weinberg equilibrium test. The outcomes of statistical analysis are presented in Table 1. Among LC patients observed genotype frequencies of some of evaluated single nucleotide polymorphisms (rs6877842, rs14035, rs13078, and rs11077) were not in agreement with HWE. It may be due to genetic changes occurring in tumor tissue during carcinogenesis, for example, loss of heterozygosity as it was described previously [15–17] or accumulation of mutations that predispose the individual to larynx cancer development.

### 3.2. The Association of miRNA Processing Genes Polymorphisms and Cancer Risk

We performed an analysis comparing the prevalence of polymorphic variants (heterozygotes or polymorphic homozygotes versus wild type homozygotes) of selected miRNA processing genes in healthy subjects and patients with LC (Table 2).

It showed that* DROSHA* rs6877842 SNP is not associated with higher occurrence of LC. We also evaluated a connection between LC and two SNPs of* DGCR8* gene: rs3757, rs417309, and rs1640299. We did not find any statistically significant connection between LC and any of rs3757 as well as rs1640299 polymorphic variants. GG genotype of* DGCR8* rs41709 is less common in LC subjects (OR: 0.3554, 0.95 CI: 0.1401–0.9015, and *p* = 0.024484). After using Bonferroni correction, this association was no longer statistically significant [*p*_corr_ = 0.220]. Similar trend of lower frequency in patients' group of CT heterozygote was observed in case of* RAN* rs14035 polymorphism (OR: 0.3158, 0.95 CI: 0.1876–0.5317, and *p* < 0.0001), and after the Bonferroni correction, the positive association remained [*p*_corr_ = 0.001]. On the other hand, the occurrence of* XPO5* rs11077 of both GT and TT polymorphic variants was higher in LC individuals than in control subjects (OR: 4.4441, 0.95 CI: 2.4723–7.9883, and *p* < 0.0001 and OR: 3.3394, 0.95 CI: 1.7544–6.3563, and *p* = 0.000183; resp.), and after the Bonferroni correction, the positive association remained [*p*_corr_ = 0.001 and *p*_corr_ = 0.002]. Also both investigated polymorphisms of* DICER1* seem to be associated with higher risk of LC occurrence (OR: 2.9762, 0.95 CI: 1.0473–8.4579, and *p* = 0.034697 [*p*_corr_ = 0.312] for TT genotype of rs13078 as well as OR: 2.6593, 0.95 CI: 1.5326–4.6144, and *p* = 0.0004 [*p*_corr_ = 0.004] for AG genotype of rs3742330). We also found a strong association between the AG and GG genotypes of the* TARBP2* rs784567 polymorphism and the risk for LC (OR: 3.0702, 0.95 CI: 1.2935–7.2871, and *p* = 0.008335 [*p*_corr_ = 0.075] and OR: 12.1429, 0.95 CI: 4.661–31.6345, and *p* < 0.0001 [*p*_corr_ = 0.001], resp.).

Secondly, we subdivided patients group according to TNM classification. In Tables 3(a) and 3(b), the correlations between the investigated gene polymorphisms and the stage of LC in comparison to healthy subjects were performed. In T1 stage we found a decreased prevalence of AG and GG genotype of* DGCR8* rs417309 (OR: 0.087, 0.95 CI: 0.0136–0.5564, and *p* = 0.011931 [*p*_corr_ = 0.107] and OR: 0.0517, 0.95 CI: 0.0098–0.2719, and *p* = 0.001265 [*p*_corr_ = 0.011], resp., Table 3(a)) as well as* RAN* rs14035 CT polymorphic variant (OR: 0.2058, 0.95 CI: 0.0415–1.0221, and *p* = 0.038382 [*p*_corr_ = 0.345], Table 3(a)). On the other hand, we found an association between T2 in LC patients and high abundance of* XPO5* rs11077 GT heterozygote (OR: 4.9697, 0.95 CI: 1.2546–19.686, and *p* = 0.016786 [*p*_corr_ = 0.150], Table 3(a)). The most interesting here was a fact of a very big difference between allele distribution in cancer and control subjects during analysis of* TARBP2* rs784567 polymorphism. It occurred that GG genotype may put an individual at high risk of T2 LC (OR: 13.3333, 0.95 CI: 1.556–114.2517, and *p* = 0.005146 [*p*_corr_ = 0.046], Table 3(a)). Analysis of subjects with T3 stage showed a decrease of CT heterozygote occurrence of* RAN* rs14035 polymorphism (OR: 0.2401, 0.95 CI: 0.1133–0.509, and *p* < 0.0001 [*p*_corr_ = 0.001], Table 3(b)) in LC patients compared to healthy subjects. Further investigation showed also a high abundance of two* XPO5* rs11077 genotypes: GT (OR: 4.8455, 0.95 CI: 2.1424–10.9591, and *p* < 0.0001 [*p*_corr_ = 0.001], Table 3(b)) and TT (OR: 3.8588, 0.95 CI: 1.5918–9.3545, and *p* = 0.001873 [*p*_corr_ = 0.017], Table 3(b)). Similar association was found related to AG* DICER1* rs3742330 heterozygote (OR: 2.2242, 0.95 CI: 1.0641–4.649, and *p* = 0.030873 [*p*_corr_ = 0.277], Table 3(b)) and GG* TARBP2* rs7834567 homozygote (OR: 7.9167, 0.95 CI: 2.3694–26.4517, and *p* = 0.00027 [*p*_corr_ = 0.002], Table 3(b)).The heterozygote of* RAN* rs14035 SNP was also found to be negatively associated with T4 (OR: 0.3902, 0.95 CI: 0.1854–0.8216, and *p* = 0.011477 [*p*_corr_ = 0.103], Table 3(b)). On the opposite side, patients with T4 stage of LC were shown to be the carriers of AG* DICER1* rs3742330 genotype (OR: 3.0583, 0.95 CI: 1.3452–6.9527, and *p* = 0.005805 [*p*_corr_ = 0.052], Table 3(b)) and* TARBP2* polymorphic variants (OR: 7.6754, 0.95 CI: 1.0036–58.6983, and *p* = 0.022201 [*p*_corr_ = 0.199] for AG and OR: 36.6667, 0.95 CI: 4.6001–292.2647, and *p* < 0.0001 [*p*_corr_ = 0.001] for GG, Table 3(b)) more often than control subjects.

SNP and lymph node status associations are presented in Table 4.* RAN* rs14035 heterozygote carriers were less likely to have both node positive or negative tumors (OR: 0.3955, 0.95 CI: 0.2264–0.6909, and *p* = 0.0009 [*p*_corr_ = 0.008] and OR: 0.131, 0.95 CI: 0.0431–0.3977, and *p* < 0.0001 [*p*_corr_ = 0.0009], resp.). Performed analysis indicated also that* XPO5* rs11077 polymorphic variants are associated with tumors without lymph node metastases (OR: 4.2177, 0.95 CI: 2.1965–8.099, and *p* < 0.0001 [*p*_corr_ = 0.001] for GT and OR: 3.5542, 0.95 CI: 1.7534–7.2043, and *p* = 0.000311 [*p*_corr_ = 0.003] for TT). Similar correlation was shown also related to* DICER1* rs13078 TT genotype (OR: 3.6111, 0.95 CI: 1.0503–12.416, and *p* = 0.033112 [*p*_corr_ = 0.298]),* DICER1* rs3742330 AG heterozygote (OR: 2.6041, 0.95 CI: 1.4276–4.7502, and *p* = 0.001483 [*p*_corr_ = 0.013]), and* TARBP2* rs784567 GG homozygote (OR: 8.5714, 0.95 CI: 3.2371–22.6961, and *p* < 0.0001 [*p*_corr_ = 0.001]).* XPO5* rs11077 and* DICER1* rs3742330 heterozygotes were also more likely to have node positive tumors (OR: 5.0584, 0.95 CI: 1.9743–12.9602, and *p* = 0.000323 [*p*_corr_ = 0.003] and OR: 3.5476, 0.95 CI: 1.3057–9.6388, and *p* = 0.009116 [*p*_corr_ = 0.082], resp.). DGCR8 rs1640299 polymorphic variants were also correlated with N0 LC stage (OR: 4.4602, 0.95 CI: 1.6527–12.0368, and *p* = 0.0016 [*p*_corr_ = 0.014] for GT and OR: 32.787, 0.95 CI: 11.1638–96.2956, and *p* < 0.0001 [*p*_corr_ = 0.001] for TT). Moreover, such association was also observed in patients with lymph node metastasis positive tumors (OR: 8.5269, 0.95 CI: 1.0874–66.8658, and *p* = 0.0159 [*p*_corr_ = 0.143] for GT and OR: 64.812, 0.95 CI: 8.0116–524.3237, and *p* < 0.0001 [*p*_corr_ = 0.0009] for TT). All investigated polymorphisms were not significantly associated with lymph node metastases when we compared groups having lymph node metastases with LC group without them.

We also analyzed the relation between miRNA processing genes polymorphic variants and the stage of cancer (Tables 5(a) and 5(b)). Some of them were associated with higher prevalence of LC in particular stage of the disease. It was found that* DICER1* rs3742330 AG heterozygote is connected with LC Stages III and IV (OR: 2.3243, 0.95 CI: 1.0828–4.9893, and *p* = 0.0276 [*p*_corr_ = 0.248] and OR: 2.9971, 0.95 CI: 1.4173–6.3378, and *p* = 0.0031 [*p*_corr_ = 0.028], resp., Table 5(b)) and* DICER1* rs13078 TT genotype is associated with LC Stage III (OR: 9.1667, 0.95 CI: 1.1122–75.5513, and *p* = 0.01450 [*p*_corr_ = 0.131], Table 5(b)). Moreover,* TARBP2* rs784567 AG heterozygote is connected to higher occurrence of LC Stage IV (OR: 9.8246, 0.95 CI: 1.2953–74.5144, and *p* = 0.0075 [*p*_corr_ = 0.068], Table 5(b)) and GG polymorphic variant is related to both LC Stage III and Stage IV (OR: 6, 0.95 CI: 1.9403–18.5537, and *p* = 0.0009 [*p*_corr_ = 0.008] and OR: 3.3333, 0.95 CI: 5.4718–343.1724, and *p* < 0.0001 [*p*_corr_ = 0.001], resp., Table 5(b)).* XPO5* rs11077 GT and TT genotypes are also associated with LC Stage III (OR: 4.9697, 0.95 CI: 2.1259–11.6175, and *p* < 0.0001 [*p*_corr_ = 0.001] and OR: 4.2876, 0.95 CI: 1.7271–10.644, and *p* = 0.001 [*p*_corr_ = 0.009], resp., Table 5(b)) and LC Stage IV (OR: 4.1573, 0.95 CI: 1.9643–8.799, and *p* = 0.0001 [*p*_corr_ = 0.0009] and OR: 2.5973, 0.95 CI: 1.1054–6.1026, and *p* = 0.0254 [*p*_corr_ = 0.221], resp., Table 5(b)).

On the other hand, performed analysis shows also that some of the investigated polymorphic variants are less common in patients with LC than in healthy people. Such phenomena were observed in patients with Stage III LC and Stage IV, where, respectively,* DGCR8* rs417309 AG and GG genotypes (OR: 0.087, 0.95 CI: 0.0136–0.5564, and *p* = 0.0119 [*p*_corr_ = 0.107] and OR: 0.0517, 0.95 CI: 0.0098–0.2719, and *p* = 0.0012 [*p*_corr_ = 0.011], Table 5(b)) as well as rs1640299 GT heterozygote (OR: 0.0968, 0.95 CI: 0.0165–0.5686, and *p* = 0.0112 [*p*_corr_ = 0.101], Table 5(b)) were less likely to be found in cancer subjects than in control group.* RAN* rs14035 CT polymorphic variant was also associated with decreased prevalence in LC patients with Stage I (OR: 0.2058, 0.95 CI: 0.0415–1.0221, and *p* = 0.0383 [*p*_corr_ = 0.345], Table 5(a)), Stage III (OR: 0.2476, 0.95 CI: 0.1166–0.5262, and *p* = 0.0001 [*p*_corr_ = 0.0009], Table 5(b)), and Stage IV (OR: 0.3478, 0.95 CI: 0.1708–0.7081, and *p* = 0.0028 [*p*_corr_ = 0.025], Table 5(b)).

### 3.3. miRNA Processing Genes Polymorphisms in relation to Smoking Status

We performed stratified analysis to estimate the interaction between miRNA processing genes single nucleotide polymorphisms and cigarette smoking (Tables  6–8, Supplementary Materials available online at http://dx.doi.org/10.1155/2015/298378). We divided patients into subgroups based on their smoking status: duration of smoking habit (nonsmokers, subject who have been smoking for less than 10 years, subjects who have been smoking in range between 20 and 40 years, and those who have been smoking for more than 40 years) as well as number of cigarettes smoked per day (nonsmokers, subjects who smoke less than 20 cigarettes daily, and those who smoke more than 20 cigarettes a day). We did not observe any elevated frequency of studied SNPs polymorphic variants correlating with cigarette smoking among LC patients in any of investigated cases. The relationship between smoking habits and susceptibility of LC occurrence in some cases was impossible to calculate due to low frequency of the genotypes.

## 4. Discussion

In this case-control study of 135LC patients and 170 cancer-free controls in a Polish population, we investigated the associations between SNPs of miRNA biosynthesis genes and risk of LC. This is the first report considering the association of the risk of larynx cancer and SNPs of the following polymorphisms:* DROSHA* (rs6877842),* DGCR8* (rs3757, rs417309, and rs1640299),* RAN* (rs14035),* XPO5* (rs11077),* DICER1* (rs13078 and rs3742330), and* TARBP2* (rs784567). The rs3742330 AG and rs13078 TT genotypes of* DICER1* are correlated with increased risk of larynx cancer. In turn, the rs14035* RAN* CT heterozygote and* DGCR8* rs417309 GG genotype were significantly inversely associated with the presence of larynx cancer. In addition, the rs3742330 of* DICER1*, rs784567 of* TARBP2*, rs417309 of* DGCR8*, and rs14035 of* RAN* as well as rs11077 of* XPO5* are associated with the LC progression depending on the tumor size. Furthermore, the* DGCR8* rs1640299,* DICER1* rs3742330 and rs13078,* RAN* rs14035, and* XPO5* rs11077 as well as rs784567 of* TARBP2* genes single nucleotide polymorphisms have demonstrated an association with tumor progression depending on the lymph node metastases. The observed genotype frequencies of rs6877842 and rs417309 alleles were not in agreement with HWE. The above information about genotype and allele frequencies of rs6877842 and rs417309 is consistent with NCBI data that shows that rs6877842 C allele frequency is about 0.018 and rs417309 A allele frequency is lower than 0.08 in population of European descent (NCBI SNP database, access date: January 13th 2015) [18, 19].

Our findings suggest, for the first time, that potentially functional polymorphisms of genes encoding proteins of miRNA processing may play a role in the tumors arising at larynx. The rs6877842 and the rs784567 polymorphisms are located in the promoter of* DROSHA* and* TRBP2* genes, respectively, and hence can affect the level of protein expression. The remaining genes polymorphisms are located in the 3′-UTR, which is the binding site of miRNAs; thereby they may affect the efficiency of miRNA processing [20–23].

Our study shows that carriers of evaluated genotypes of miRNAs processing genes may be put at higher risk of larynx cancer development with high probability of lymph nodes occurrence. On the other hand, it appeared that RAN rs14035 CT polymorphic variant may possess a kind of protective effect on individuals, because a relatively small number of LC patients were carriers of this heterozygote, even in comparison with healthy subjects. To the best of our knowledge, polymorphisms evaluated in our study were not analyzed in the context of larynx cancer before. There is also a very little information about the connection of investigated genes with head and neck cancer. On the other hand, SNPs of miRNAs processing genes are widely analyzed in other types of cancer; however, these pieces of data are inconsistent.

In the present association study, we found that* DROSHA* polymorphism was not associated with the risk of larynx cancer. DROSHA is a member of RNase III superfamily and is an important nuclease that executes the initial step in miRNA processing by transforming pri-miRNA to pre-miRNA. RNA interference of DROSHA resulted in accumulation of pri-miRNA and reduction of pre-miRNA and mature RNA [24]. There were some studies describing the role of* DROSHA* in cancer [25]. We have found only few papers investigating role of* DROSHA* rs6877842 SNP in T-cell lymphoma [26, 27], esophageal cancer [28], and idiopathic ovarian insufficiency [29]. Tian et al.'s data showed that among patients with T-cell lymphoma carriers of GC genotype in Chinese population have higher complete remission rate compared with those carrying CC genotype (OR: 0.07, 0.95 CI: 0.01–0.072, and *p* = 0.026) [26]. It was also confirmed by Li et al. who proved that variant allele of this polymorphism also increased the overall survival of T-cell lymphoma patients compared to the wild type genotype (HR: 0.27, 0.95 CI: 0.11–0.67, and *p* = 0.005) [19]. Additionally, a haplotype analysis of* DROSHA* rs6877842 in Korean patients suggests that ^*∗∗∗*^ACTA is associated with higher POI [13]. In case of esophageal cancer there was no connection between prevalence of investigated polymorphism and tumor occurrence. Although there is no information about connection between head and neck cancers and rs6877842, there are some proofs of dysregulation of miRNA processing genes at the expression level. Guo et al. showed the mean level of DROSHA and DICER mRNA was significantly downregulated in nasopharyngeal cancer (NPC) tissue specimens and cell lines when compared with controls. Low expression of DICER and DROSHA protein was significantly correlated with shorter progression-free survival and overall survival of NPC patients [30].

DICER1 is an enzyme responsible for the cleavage of miRNA precursors and has previously been implicated in the oncogenic process of several cancers [31–33]. DICER1 and transactivation-responsive RNA-binding protein mediate pre-miRNA processing. A recent study indicated that DICER1 functions as a haploinsufficient tumor suppressor in cancer [34]. Indeed, lower levels of* DICER1* mRNA have been associated with decreased cancer survival [35]. Evidence indicates that* DICER1* may play crucial roles in the tumorigenesis of different cancers. Some studies showed that lower levels of* DICER1* mRNA expression were associated with the development of lung cancer [36], colon cancer [33], and ovarian cancer [35]. However, studies also demonstrated that elevated expression levels of* DICER1* were correlated with increased cell proliferation of oral cancer cells [37]. Moreover, Gao and colleagues showed that the expression of DICER was significantly higher in the laryngeal squamous cell carcinoma (LSCC) than in the polyp tissue specimens. DICER expression level was significantly associated with the TNM stage. Survival analyses also revealed a strong association between tumor DICER expression and the survival of the patients with LSCC [38]. On the other hand, as it was mentioned before, Guo et al.'s study showed decreased mRNA expression in nasopharyngeal cancer [30]. These analyses show that upregulation or downregulation of DICER mRNA expression may depend on the site of tumor appearance. We have found an association between risk of laryngeal cancer and two DICER1 SNPs: rs13078 and rs3742330. It was also found that rs13078 (HR = 1.66; 0.95 CI: 1.09–2.52; *p* = 0.02) was associated with the risk of death of patients with colorectal adenocarcinoma [39]. Lin et al. performed an analysis of correlation between survival and recurrence in patients with renal cell carcinoma and* DICER* SNPs [40]. In haplotype analysis, haplotypes of* DICER* showed significant association with RCC survival. Specifically, compared with the AT haplotype (in order of rs3742330 and rs13078), the haplotype AA had HR of 1.51 (95% CI = 0.99–2.31) and the haplotype GA was associated with increased HR of 2.04 (95% CI = 1.00–4.15). Similarly, in diplotype analysis, using the AT-AT as the reference group, the diplotype AT-GT was associated with 2.86-fold increased risk (95% CI = 1.11–7.34; *p* = 0.03) and the diplotype AA-AA was at 3.48-fold increased risk (95% CI = 1.21–9.97; *p* = 0.02). Another study showed that patients with at least one variant allele of SNP rs3742330 in* DICER* had a significantly increased risk of oral premalignant lesions (OR, 2.09; 95% CI, 1.03–4.24) [41]. On the other hand, there was no statistically significant association between rs3742330 and rs13078 SNPs and esophageal cancer [28].

DGCR8 is a double-stranded RNA-binding protein that functions as the noncatalytic subunit of the microprocessor complex and facilitates RNA cleavage by the RNase III protein DROSHA.* In vitro* knockdown of* DROSHA*,* DGCR8*, and* DICER1* impaired miRNA processing and thereby promoted oncogenic transformation in mouse lung cancer cells and tumor development* in vivo* [42]. Han et al. [43] reported that* RNASEN* and* DGCR8* regulate each other posttranscriptionally and that* DGCR8* stabilizes* RNASEN* via protein-protein interactions. Because of the direct effect of* DGCR8* and* RNASEN* on miRNA biogenesis and the associations between miRNA expression and cancer development the variations in either gene might affect head and neck cancer occurrence. In our study we evaluated the role of three* DGCR8* SNPs in larynx cancer: rs3757, rs417309, and rs1640299. Other researchers also evaluated the role of these polymorphisms in context of renal cell carcinoma and esophageal cancer. Horikawa et al. and Lin et al. also evaluated these polymorphisms referred to renal cell carcinoma, but SNPs as well as haplotype analysis did not reveal any statistically significant correlations [40, 44]. Study performed by Ye et al. also did not show any association between these polymorphisms and esophageal cancer [28]. No linkage was also presented between the occurrence of cervical cancer and rs3757 polymorphic variants [45]. However, Jiang et al. have shown that the rs417309 polymorphism of* DGCR8* gene was associated with an increased breast cancer risk (OR = 1.50; 95% confidence interval (CI): 1.16–1.93). Besides, using luciferase assay, they have found that the variant A allele of rs417309 compared to allele G elevates DGCR8 protein expression [46]. Because the rs417309 polymorphism is located in the 3′-UTR, which is the binding site of miR-106b and miR-579, it might affect the miRNAs maturation. Gong et al. have indicated that the expression of miR-106b was dramatically increased in breast cancer tissues compared to in healthy tissue [47]. Additionally, it was shown that the expression of* DGCR8* was upregulated in several types of cancer [48, 49]. Therefore, rs417309 may impair the binding of miRNAs such as miR-106b with DGCR8 and disrupt the process of miRNAs maturation and consequently play an important role in the tumorigenesis.

RAN is a unique member of the Ras superfamily of GTPases, which is essential to the transportation of pre-miRNAs from nucleus to cytoplasm through the nuclear pore complex in a GTP-dependent manner [50]. PolymiRTS database suggests that the ancestral allele lies in a binding site for miR-575, which is disrupted by the derived allele that in addition creates a binding site for miR-182^*∗*^. Although these are* in silico* results, they raise the possibility that in addition to affecting cancer risk through the disruption of miRNA nuclear export a more intricate pathway may be involved that includes miRNA regulation. Some studies have investigated the associations between rs14035 polymorphism of this gene and risk of several cancers. Evaluation of role of rs14035 in hepatocellular carcinoma showed no statistically significant differences (*p* < 0.05) between patients and healthy controls [51]. There was also no association with the risk of renal cell carcinoma [44] or oral premalignant lesions [52]. On the other hand, data showed an association between occurrence of recessive variant of investigated RAN polymorphism and esophageal cancer (*p* = 0.024) [28]. rs14035 was also evaluated as a predictor of clinical outcomes in colorectal adenocarcinoma patients [34]. Among 117 patients with Stage II disease who received 5-FU based chemotherapy, the most significant association with recurrence was conferred by the variant allele of RAN:rs14035 in a dose-dependent manner (per allele HR = 2.32; 95% CI, 1.28 to 4.21; *p* = 0.005; *q* = 0.06). For Stage II diseases, RAN:rs14035 was associated with overall survival with high significance in patients receiving surgery and adjuvant fluoropyrimidine treatment.

Xpo5 mediates nuclear export of pre-miRNA in a RAN GTP-dependent manner by binding to pre-miRNA and RAN GTPase in the nucleus [53]. XPO5 is found in the nuclear membrane and mediates the transport of pre-miRNA between the nucleic and cytoplasmic compartments so as to adjust the whole miRNA expression level. Knock-down of XPO5 expression leads to reduced miRNA levels [53]. A mutated and inactive XPO5 resulted in reduced miRNA processing and decreased miRNA target inhibition; the restored XPO5 seemed as a tumor suppressor to reverse the impaired export of pre-miRNA in colon cancer [54]. The miR-SNP of rs11077 of XPO5 has been associated with the risk of esophageal cancer (OR = 1.84, 95% CI: 1.16–2.93, and *p* = 0.010) as well as the overall survival in myeloma and lymphoma [28, 55, 56]. The AC genotype of rs11077, which carries C or A allele, was significantly associated with a better chemotherapy response in patients with non-small cell lung (*p* = 0.001). In addition, rs11077 was independently associated with overall survival in advanced NSCLC patients through multivariate analysis (relative risk 0.457; 95% confidence interval: 0.251–0.831; *p* = 0.010) [57]. The altered* XPO5* expression may affect the miRNAs, leading to overall downregulation of miRNA expression profiles and thereby mediates the hepatocellular carcinoma survival. rs11077 CC genotype shows association with reduced Renilla expression in a Renilla luciferase 3′UTR reporter system. It implies that this SNP could modify* XPO5* expression so as to result in overall expression of miRNA in multiple myeloma cells [35].

TARBP2 (trans-activation-responsive RNA binding protein 2) is a component of the miRNA loading complex (composed of DICER1, AGO2, and TRBP2) required for the formation of RISC. Melo and colleagues [58] identified two frameshift mutations in TARBP2 that introduce premature stop codons, resulting in reduced TRBP expression. One function of TRBP is regulating DICER1 stability; thus these mutations resulted in reduced* DICER1* expression and lower miRNA production and were associated with higher cellular proliferation levels [58]. It has been shown that the variant allele of rs784567, which is located in the promoter of the* TRBP* gene, was associated with neither a risk of bladder cancer (*p* = 0.07) [21] nor renal cell carcinoma [40] or oral premalignant lesions [41]. Patients with Hodgkin Lymphoma that were carriers of both* XPO5* AA/CC and* TARBP2* TT/TC genotypes had the shortest disease free survival (*p* = 0.008) and overall survival (*p* = 0.008). On the other hand, rs784567 (HR = 1.59; 95% CI, 1.03 to 2.43; *p* = 0.04) was also associated with the risk of death in colorectal cancer but lost significance after adjusting for multiple comparison [33]. Given the differential cell of origin for cancers and differential cell type specificity of miRNA transcriptomes, it is reasonable to assume that the effects of miR-SNP will be modulated in a cell type-specific manner.

Several potential limitations of the present study warrant considerations. First of all, a relatively small sample size may limit the statistical power of our study, especially in stratification analysis by tumor sites. Secondly, it is a hospital-based case-control study and inherent selection bias cannot be completely excluded. Thirdly, since the intensity and duration of drinking were absent in this study, it was difficult to do future analysis for such exposure variables. Thus, larger, well-designed epidemiological studies with ethnically diverse populations are warranted to confirm and expand our findings.

However, our findings provide new information about the relationship of genes involved in the microRNAs maturation and the development of larynx cancer. The results indicate that polymorphic variants of these genes may affect not only the development of cancer but also disease progression. Further studies confirming our results with larger study group can help to discover new diagnostic markers of LC, which greatly facilitate the initiation of treatment and consequently a better prognosis for the patient. Furthermore, our results may also have a large impact on the clinical studies, because they provide information about cancer progression.

## 5. Conclusion

In light of recent reports, the role of microRNAs in cancer development is considerable. The miRNAs are involved in all aspects of cancer biology, such as proliferation, apoptosis, migration, and angiogenesis. Our results suggest that rs3742330 of* DICER1*, rs13078 of* DICER1*, and rs784567 of* TARBP2* as well as rs11077 of* XPO5* might be associated with a risk of laryngeal cancer occurrence in the Polish population. Additionally, rs417309 of* DGCR8*, rs3742330 and rs13078 of* DICER1*, and rs784567 of* TARBP2* as well as rs11077 of* XPO5* are associated with the progression of LC depending on the tumor size and lymph node metastases. However, further epidemiologic studies with larger subject numbers should be performed to confirm and expand our results. In addition, the results of our study warrant further functional studies to elucidate the mechanisms by which polymorphisms of miRNA machinery genes affect LC development.

## Supplementary Material

The Tables 6–8 provide an assessment of association between miRNA processing genes single nucleotide polymorphisms and smoking cigarettes. Table 6 shows the relationship of these SNPs with smoking status, Table 7 with duration of smoking, while Table 8 number of cigarettes smoked per day. Performed analysis did not show any statistically significant differences in the frequency of studied SNPs polymorphic variants correlating with cigarette smoking among LC patients.

## Figures and Tables

**Table 1:** The genotype frequencies for each evaluated polymorphism using the Hardy-Weinberg equilibrium test.

Gene/rs number	Chi-squared test *p* value for patients group	Chi-squared test *p* value for control group
*DROSHA*/6877842	0.537047	0.384044
*DICER1*/3742330	0.000000	0.000001
*DICER1*/13078	0.987719	0.000000
*DGCR8*/1640299	0.021333	0.406734
*DGCR8*/3757	0.000000	0.000000
*DGCR8*/417309	0.026576	0.227311
*RAN*/14035	0.540760	0.002413
*TARBP2*/784567	0.006552	0.000005
*XPO5*/11077	0.941889	0.000001

**Table 2:** The odds ratio (OR) of the miRNA processing genes single nucleotide polymorphisms in head and neck cancer patients and healthy controls.

Gene/rs number	Genotype	Controls number	Patients number	Odds ratio	0.95 confidence intervals		*p*
					(0.95 CI)		
					Lower limit	Upper limit	
*DROSHA*							
6877842	CC	15	6	Ref.			
	CG	79	49	1.5506	0.5638	4.2644	0.392883
	GG	76	73	2.4013	0.8836	6.526	0.078775

*DICER1*							
3742330	AA	63	23	Ref.			
	AG	103	100	2.6593	1.5326	4.6144	0.0004
	GG	4	0	NA			
13078	AA	10	7	Ref.			
	AT	124	46	0.53	0.1904	1.4747	0.17013F
	TT	36	75	2.9762	1.0473	8.4579	0.034697

*DGCR8 *							
1640299	GG	16	6	Ref.			
	GT	93	47	1.3477	0.4949	3.6696	0.559829
	TT	61	60	2.623	0.9614	7.1562	0.053443
3757	AA	15	4	Ref.			
	AG	119	89	2.8046	0.9	8.74	0.065196
	GG	36	29	3.0208	0.9039	10.0951	0.064411
417309	AA	8	13	Ref.			
	AG	46	32	0.4281	0.1591	1.1516	0.088032
	GG	116	67	0.3554	0.1401	0.9015	0.024484

*RAN*							
14035	CC	67	73	Ref.			
	CT	93	32	0.3158	0.1876	0.5317	<0.0001
	TT	10	5	0.4589	0.1492	1.4115	0.165857

*TARBP2*							
784567	AA	35	7	Ref.			
	AG	114	70	3.0702	1.2935	7.2871	0.008335
	GG	21	51	12.1429	4.661	31.6345	<0.0001

*XPO5*							
11077	GG	82	26	Ref.			
	GT	44	62	4.4441	2.4723	7.9883	<0.0001
	TT	34	36	3.3394	1.7544	6.3563	0.000183

**(a):** 

Gene/rs number	Genotype	Controls number	T1					T2				
			Patients	Odds ratio	0.95 confidence intervals		*p*	Patients	Odds ratio	0.95 confidence intervals		*p*
			number		Lower limit	Upper limit		number		Lower limit	Upper limit	
*DROSHA*												
6877842	CC	15	1	Ref.				0	Ref.			
	CG	79	5	0.9494	0.1034	8.7141	0.6590^F^	6	NA			
	GG	76	4	0.7595	0.0793	7.2769	0.5940^F^	11	NA			

*DICER1*												
3742330	AA	63	1	Ref.				3	Ref.			
	AG	103	8	4.8932	0.5978	40.0546	0.0967^F^	12	2.4466	0.6645	9.0082	0.1669
	GG	4	0	NA				0	NA			
13078	AA	10	0	Ref.				1	Ref.			
	AT	124	8	NA				8	0.6452	0.0732	5.6871	0.5239^F^
	TT	36	2	NA				8	2.2222	0.2478	19.9312	0.4181^F^

*DGCR8 *												
1640299	GG	16	0	Ref.				1	Ref.			
	GT	93	2	NA				9	1.5652	0.1854	13.2126	0.5614^F^
	TT	61	4	NA				5	1.3115	0.1429	12.0333	0.6432^F^
3757	AA	15	1	Ref.				0	Ref.			
	AG	119	7	0.8824	0.1014	7.6744	0.6255^F^	15	NA			
	GG	36	1	0.4167	0.0244	7.107	0.5166^F^	2	NA			
417309	AA	8	4	Ref.				2	Ref.			
	AG	46	2	0.087	0.0136	0.5564	0.0119^F^	8	0.6957	0.1243	3.892	0.4909^F^
	GG	116	3	0.0517	0.0098	0.2719	0.0012^F^	5	0.1724	0.0288	1.0322	0.0898^F^

*RAN*												
14035	CC	67	7	Ref.				9	Ref.			
	CT	93	2	0.2058	0.0415	1.0221	0.0383^F^	6	0.4803	0.1632	1.4139	0.1761
	TT	10	1	0.9571	0.1063	8.6223	0.7245^F^	0	NA			

*TARBP2*												
784567	AA	35	1	Ref.				1	Ref.			
	AG	114	6	1.8421	0.2144	15.8246	0.4907^F^	8	2.4561	0.2969	20.3221	0.3494^F^
	GG	21	2	3.3333	0.2846	39.0437	0.3346^F^	8	13.3333	1.556	114.2517	0.0051^F^

*XPO5*												
11077	GG	82	2	Ref.				3	Ref.			
	GT	44	4	3.7273	0.6565	21.1609	0.1274^F^	8	4.9697	1.2546	19.686	0.0167^F^
	TT	34	3	3.6176	0.5784	22.6277	0.1663^F^	5	4.0196	0.9094	17.7671	0.0634^F^

^F^ *p* values calculated with Fisher's exact probability test.

**(b):** 

Gene/rs number	Genotype	T3						T4					
		Controls	Patients	Odds ratio	0.95 confidence intervals		*p*	Controls	Patients	Odds ratio	0.95 confidence intervals		*p*
		number	number		Lower limit	Upper limit		number	number		Lower limit	Upper limit	
*DROSHA*													
6877842	CC	15	4	Ref.				15	1	Ref.			
	CG	79	17	0.807	0.238	2.7364	0.4730^F^	79	21	3.9873	0.4978	31.9386	0.1440^F^
	GG	76	32	1.5789	0.4863	5.127	0.4424	76	26	5.1316	0.6458	40.776	0.0747^F^

*DICER1*													
3742330	AA	63	11	Ref.				63	8	Ref.			
	AG	103	40	2.2242	1.0641	4.649	0.0308	103	40	3.0583	1.3452	6.9527	0.0058
	GG	4	0	NA				4	0	NA			
13078	AA	10	3	Ref.				10	3	Ref.			
	AT	124	15	0.4032	0.0997	1.6304	0.1859^F^	124	15	0.4032	0.0997	1.6304	0.1859^F^
	TT	36	35	3.2407	0.8222	12.7733	0.0807	36	30	2.7778	0.7001	11.0216	0.1345

*DGCR8*													
1640299	GG	16	2	Ref.				16	3	Ref.			
	GT	93	23	1.978	0.4245	9.2218	0.3022^F^	93	13	0.7455	0.1908	2.9131	0.4531
	TT	61	27	3.541	0.7605	16.4881	0.0739	61	24	2.0984	0.5603	7.8588	0.2067
3757	AA	15	3	Ref.				15	0	Ref.			
	AG	119	34	1.4286	0.3905	5.2255	0.4235^F^	119	34	NA			
	GG	36	15	2.0833	0.5251	8.2662	0.2317^F^	36	11	NA			
417309	AA	8	3	Ref.				8	4	Ref.			
	AG	46	13	0.7536	0.1745	3.2541	0.4838^F^	46	9	0.3913	0.0968	1.5817	0.1704^F^
	GG	116	28	0.6437	0.1604	2.5833	0.3825^F^	116	31	0.5345	0.151	1.8918	0.2557^F^

*RAN*													
14035	CC	67	33	Ref.				67	24	Ref.			
	CT	93	11	0.2401	0.1133	0.509	<0.0001	93	13	0.3902	0.1854	0.8216	0.0114
	TT	10	3	0.6091	0.157	2.3634	0.3528^F^	10	1	0.2792	0.0339	2.2978	0.1914^F^

*TARBP2*													
784567	AA	35	4	Ref.				35	1	Ref.			
	AG	114	31	2.3794	0.7857	7.2059	0.1160	114	25	7.6754	1.0036	58.6983	0.0222
	GG	21	19	7.9167	2.3694	26.4517	0.00027	21	22	36.6667	4.6001	292.2647	<0.0001

*XPO5*													
11077	GG	82	10	Ref.				82	11	Ref.			
	GT	44	26	4.8455	2.1424	10.9591	<0.0001	44	24	4.0661	1.8231	9.0689	0.000364
	TT	34	16	3.8588	1.5918	9.3545	0.001873	34	12	2.631	1.0584	6.5403	0.033306

^F^ *p* values calculated  with Fisher's exact probability test.

**Table 4:** An association of the miRNA processing genes single nucleotide polymorphisms with lymph node metastases occurrence in head and neck cancer.

Gene/rs number	Genotype	Controls number	N0					N1–3					N1–3 versus N0			
			Patients	Odds ratio	0.95 confidence intervals		*p*	Patients	Odds ratio	0.95 confidence intervals		*p*	Odds ratio	0.95 confidence intervals		*p*
			Number		Lower limit	Upper limit		number		Lower limit	Upper limit			Lower limit	Upper limit	
*DROSHA*																
6877842	CC	15	6	Ref.				0	Ref.				Ref.			
	CG	79	36	1.1392	0.4085	3.1769	0.8064	13	NA				NA			
	GG	76	51	1.6776	0.6104	4.611	0.3125	22	NA				NA			

*DICER1*																
3742330	AA	63	18	Ref.				5	Ref.				Ref.			
	AG	103	71	2.6041	1.4276	4.7502	0.0014	29	3.5476	1.3057	9.6388	0.0091	1.4704	0.4989	4.3338	0.4839
	GG	4	0	NA				0	NA				NA			
13078	AA	10	4	Ref.				3	Ref.				Ref.			
	AT	124	38	0.7661	0.2273	2.5825	0.4394^F^	8	0.2151	0.0492	0.9401	0.0612^F^	0.2807	0.0523	1.506	0.1470^F^
	TT	36	52	3.6111	1.0503	12.416	0.0331	23	2.1296	0.5292	8.5696	0.2263^F^	0.5897	0.122	2.85	0.3904^F^

*DGCR8*																
1640299	GG	16	5	Ref.				1	Ref.				Ref.			
	GT	93	34	4.4602	1.6527	12.0368	0.0016	13	8.5269	1.0874	66.8658	0.0159	1.911	0.2035	17.9611	0.493^F^
	TT	61	43	32.787	11.1638	96.2956	<0.0001	17	64.812	8.0116	524.3237	<0.0001	1.976	0.2148	18.1876	0.474^F^
3757	AA	15	4	Ref.				0	Ref.				Ref.			
	AG	119	63	1.9853	0.6321	6.2352	0.2334	26	NA				NA			
	GG	36	23	2.3958	0.7068	8.1213	0.1532	6	NA				NA			
417309	AA	8	11	Ref.				2	Ref.				Ref.			
	AG	46	25	0.4427	0.1589	1.2335	0.1138	7	0.6087	0.1067	3.4736	0.4382^F^	1.54	0.2746	8.6355	0.4822^F^
	GG	116	49	0.3072	0.1165	0.8104	0.0131	18	0.6207	0.122	3.1588	0.4176^F^	2.0204	0.4077	10.013	0.3118^F^

*RAN*																
14035	CC	67	51	Ref.				22	Ref.				Ref.			
	CT	93	28	0.3955	0.2264	0.6909	0.0009	4	0.131	0.0431	0.3977	<0.0001	0.3312	0.1037	1.0573	0.0541
	TT	10	4	0.5255	0.1559	1.7718	0.2942	1	0.3045	0.0369	2.5153	0.2253	0.5795	0.0612	5.4857	0.5363

*TARBP2*																
784567	AA	35	7	Ref.				0	Ref.				Ref.			
	AG	114	50	2.193	0.9124	5.2711	0.0741	20	NA				NA			
	GG	21	36	8.5714	3.2371	22.6961	<0.0001	15	NA				NA			

*XPO5*																
11077	GG	82	19	Ref.				7	Ref.				Ref.			
	GT	44	43	4.2177	2.1965	8.099	<0.0001	19	5.0584	1.9743	12.9602	0.0003	1.1993	0.432	3.3294	0.7290
	TT	34	28	3.5542	1.7534	7.2043	0.0003	8	2.7563	0.9264	8.2008	0.0599^F^	0.7755	0.2407	2.4981	0.6713

^F^ *p* values calculated with Fisher's exact probability test.

**(a):** 

Gene/rs number	Genotype	Controls number	Stage I					Stage II				
			Patients	Odds ratio	0.95 confidence intervals		*p* ^*∗*^	Patients	Odds ratio	0.95 confidence intervals		*p* ^*∗*^
			number		Lower limit	Upper limit		number		Lower limit	Upper limit	
*DROSHA*												
6877842	CC	15	1	Ref.				0	Ref.			
	CG	79	5	0.9494	0.1034	8.7141	0.6590	3	NA			
	GG	76	4	0.7895	0.0824	7.5674	0.6067	5	NA			

*DICER1*												
3742330	AA	63	1	Ref.				2	Ref.			
	AG	103	8	4.8932	0.5978	40.0546	0.0967	4	1.2233	0.2177	6.8735	0.5907
	GG	4	0	NA				0	NA			
13078	AA	10	0	Ref.				1	Ref.			
	AT	124	8	NA				6	0.4839	0.0529	4.4237	0.4409
	TT	36	2	NA				1	0.2778	0.0159	4.8457	0.4095

*DGCR8*												
1640299	GG	16	0	Ref.				0	Ref.			
	GT	93	2	NA				6	NA			
	TT	61	4	NA				2	NA			
3757	AA	15	1	Ref.				0	Ref.			
	AG	119	7	0.8824	0.1014	7.6744	0.6255	6	NA			
	GG	36	1	0.4167	0.0244	7.107	0.5166	2	NA			
417309	AA	8	4	Ref.				1	Ref.			
	AG	46	2	0.087	0.0136	0.5564	0.0119	4	0.6957	0.0686	7.0533	0.5767
	GG	116	3	0.0517	0.0098	0.2719	0.0012	2	0.1379	0.0113	1.689	0.1993

*RAN*												
14035	CC	67	7	Ref.				2	Ref.			
	CT	93	2	0.2058	0.0415	1.0221	0.0383	5	1.8011	0.3392	9.5644	0.3884
	TT	10	1	0.9571	0.1063	8.6223	0.7245	0	NA			

*TARBP2*												
7845672	AA	35	1	Ref.				0	Ref.			
	AG	114	6	1.8421	0.2144	15.8246	0.4907	3	NA			
	GG	21	2	3.3333	0.2846	39.0437	0.3346	5	NA			

*XPO5*												
11077	GG	82	2	Ref.				1	Ref.			
	GT	44	4	3.7273	0.6565	21.1609	0.1274	4	7.4545	0.8082	68.7565	0.06
	TT	34	3	3.6176	0.5784	22.6277	0.1663	3	7.2353	0.7267	72.0402	0.0865

^*∗*^All *p* values are for Fisher's exact probability test due to low sample sizes.

**(b):** 

Gene/rs number	Genotype	Controls number	Stage III					Stage IV				
			Patients	Odds ratio	0.95 confidence intervals		*p*	Patients	Odds ratio	0.95 confidence intervals		*p*
			number		Lower limit	Upper limit		number		Lower limit	Upper limit	
*DROSHA*												
6877842	CC	15	4	Ref.				1	Ref.			
	CG	79	16	0.7595	0.2227	2.5903	0.4371^F^	24	4.557	0.572	36.3009	0.1034^F^
	GG	76	31	1.5296	0.4703	4.9749	0.4795	33	6.5132	0.8259	51.3637	0.0341^F^

*DICER1*												
3742330	AA	63	10	Ref.				10	Ref.			
	AG	103	38	2.3243	1.0828	4.9893	0.0276	49	2.9971	1.4173	6.3378	0.0031
	GG	4	0	NA				0	NA			
13078	AA	10	1	Ref.				4	Ref.			
	AT	124	14	1.129	0.1344	9.4878	0.6945^F^	17	0.3427	0.0967	1.215	0.1004^F^
	TT	36	33	9.1667	1.1122	75.5513	0.01450^F^	38	2.6389	0.7591	9.1733	0.1006^F^

*DGCR8*												
1640299	GG	16	2	Ref.				4	Ref.			
	GT	93	21	1.8065	0.3856	8.4634	0.3533^F^	18	0.0968	0.0165	0.5686	0.0112^F^
	TT	61	26	3.4098	0.731	15.9056	0.0836^F^	26	0.5	0.0841	2.9719	0.3691^F^
3757	AA	15	2	Ref.				0	Ref.			
	AG	119	33	2.0798	0.4526	9.5571	0.2709^F^	42	NA			
	GG	36	10	2.0833	0.4068	10.6686	0.3073^F^	11	NA			
417309	AA	8	4	Ref.				3	Ref.			
	AG	46	13	0.5652	0.1467	2.178	0.3094^F^	13	0.7536	0.1745	3.2541	0.4838^F^
	GG	116	25	0.431	0.1204	1.5436	0.1704^F^	35	0.8046	0.2025	3.1973	0.4996^F^

*RAN*												
14035	CC	67	32	Ref.				29	Ref.			
	CT	93	11	0.2476	0.1166	0.5262	0.0001	14	0.3478	0.1708	0.7081	0.0028
	TT	10	3	0.6281	0.1617	2.4405	0.3715^F^	1	0.231	0.0283	1.8892	0.1277^F^

*TARBP2*												
784567	AA	35	5	Ref.				1	Ref.			
	AG	114	28	1.7193	0.6174	4.788	0.2942	32	9.8246	1.2953	74.5144	0.0075
	GG	21	18	6	1.9403	18.5537	0.0009	26	43.3333	5.4718	343.1724	<0.0001

*XPO5*												
11077	GG	82	9	Ref.				13	Ref.			
	GT	44	24	4.9697	2.1259	11.6175	<0.0001	29	4.1573	1.9643	8.799	0.0001
	TT	34	16	4.2876	1.7271	10.644	0.001	14	2.5973	1.1054	6.1026	0.0254

^F^ *p* values calculated with Fisher's exact probability test.
