# Supplementary material for: Association of Polymorphic Variants of miRNA Processing Genes with Larynx Cancer Risk in a Polish Population
Source: Biomed Res Int. 2015 Nov 25;2015:298378. doi: 10.1155/2015/298378 (PMC4673325; doi:10.1155/2015/298378)
Supplement: Supplementary file 1 — The Tables 6–8 provide an assessment of association between miRNA processing genes single nucleotide polymorphisms and smoking cigarettes. Table 6 shows the relationship of these SNPs with smoking status, Table 7 with duration of smoking, while Table 8 number of cigarettes smoked per day. Performed analysis did not show any statistically significant differences in the frequency of studied SNPs polymorphic variants correlating with cigarette smoking among LC patients. [file 298378.f1.pdf]

Table 6. An association of the miRNA processing genes single nucleotide polymorphisms with head and neck cancer risk by smoking status.

		Non smokers	Smokers		.95 Confidence Intervals		
**GENE/ RS NO.**	**GENOTYPE**	**NO.**	**NO.**	**Odds Ratio**	**Lower Limit**	**Upper Limit**	**p***
DROSHA							
**6877842**	CC	0	6	Ref.			
	CG	8	41	NA			
	GG	3	70	NA			
DICER1							
**3742330**	AA	3	20	Ref.			
	AG	7	93	1.9929	0.474	8.3795	0.2791
	GG	0	0	NA			
**13078**	AA	0	7	Ref.			
	AT	4	42	NA	0.2983	3.9161	0.5897
	TT	7	68	NA			
DGCR8							
**1640299**	GG	0	6	Ref.			
	GT	4	43	NA			
	TT	5	55	NA			
**3757**	AA	0	4	Ref.			
	AG	7	82	NA			
	GG	3	26	NA			
**417309**	AA	1	12	Ref.			
	AG	3	29	0.8056	0.076	8.5409	0.6741
	GG	6	61	0.8472	0.0933	7.6897	0.6821
RAN							
**14035**	CC	6	67	Ref.			
	CT	1	31	2.7761	0.3203	24.0583	0.3109
	TT	1	4	0.3582	0.0343	3.7378	0.3832
TARBP2							
**784567**	AA	1	6	Ref.			
	AG	3	67	3.7222	0.3335	41.5432	0.3224
	GG	6	45	1.25	0.1276	12.2453	0.6149
XPO5							
**11077**	GG	2	24	Ref.			
	GT	6	56	0.7778	0.1464	4.1327	0.5623
	TT	3	33	0.9167	0.142	5.9173	0.6535

*All p values are for Fisher Exact Probability Test due to low sample sizes.

Table 7. An association of the miRNA processing genes single nucleotide polymorphisms with head and neck cancer risk modified by smoking duration.

			Up to 10 years					20-40 years					More than 40				
		Non smokers	PATIENTS	Odds Ratio	.95 Confidence Intervals			PATIENTS	Odds Ratio	.95 Confidence Intervals			PATIENTS	Odds Ratio	.95 Confidence Intervals		
GENE/ RS NO.	GENO TYPE	NO.	NO.		Lower Limit	Upper Limit	p*	NO.		Lower Limit	Upper Limit	p*			Lower Limit	Upper Limit	p*
DROSHA																	
**6877842**	CC	0	4	Ref.				0	Ref.				2	Ref.			
	CG	8	11	NA				15	NA				15	NA			
	GG	3	31	NA				21	NA				17	NA			
DICER1																	
**3742330**	AA	3	9	Ref.				4	Ref.				7	Ref.			
	AG	7	37	1.7619	0.3791	8.1876	0.3628	31	3.3214	0.6026	18.308	0.1722	24	0.6806	0.1383	3.3479	0.4639
	GG	0	0	NA				0	NA				0	NA			
**13078**	AA	0	1	Ref.				4	Ref.				2	Ref.			
	AT	4	16	NA				15	NA				11	NA			
	TT	7	28	NA				18	NA				21	NA			
DGCR8																	
**1640299**	GG	0	2	Ref.				3	Ref.				1	Ref.			
	GT	4	19	NA				11	NA				13	NA			
	TT	5	19	NA				18	NA				18	NA			
**3757**	AA	0	2	Ref.				1	Ref.				1	Ref.			
	AG	7	31	NA				27	NA				23	NA			
	GG	3	9	NA				8	NA				9	NA			
**417309**	AA	1	5	Ref.				5	Ref.				2	Ref.			
	AG	3	9	0.6	0.0486	7.4083	0.5931	8	0.5333	0.0427	6.6553	0.5546	11	1.8333	0.1209	27.7987	0.5794
	GG	6	19	0.6333	0.0613	6.5422	0.5869	23	0.7667	0.0748	7.86	0.6559	18	0.3636	0.0693	1.9094	0.2098
RAN																	
**14035**	CC	6	27	Ref.				19	Ref.				21	Ref.			
	CT	1	6	1.3333	0.1344	13.2251	0.6449	13	4.1053	0.4408	38.2355	0.1924	12	3.4286	0.3677	31.9713	0.2540
	TT	1	3	0.6667	0.0587	7.5722	0.5850	0	NA				1	0.0952	0.0083	1.0908	0.0627
TARBP2																	
**784567**	AA	1	5	Ref.				1	Ref.				0	Ref.			
	AG	3	24	1.6	0.1367	18.7236	0.5711	20	6.6667	0.3234	137.4111	0.2999	22	NA			
	GG	6	16	0.5333	0.0512	5.5541	0.5221	16	2.6667	0.1429	49.7588	0.5072	12	NA			
XPO5																	
**11077**	GG	2	9	Ref.				8	Ref.				7	Ref.			
	GT	6	20	0.7407	0.1245	4.4068	0.5558	19	0.7917	0.1307	4.7939	0.5888	17	0.8095	0.1303	5.0285	0.6018
	TT	3	12	0.8889	0.1219	6.4834	0.6543	9	0.75	0.0988	5.693	0.5939	10	0.9524	0.1247	7.2755	0.6842

*All p values are for Fisher Exact Probability Test due to low sample sizes.

Table 8. An association of the miRNA processing genes single nucleotide polymorphisms with head and neck cancer risk modified by number of cigarettes smoked per day.

			Up to 20 cigarettes per day					More than 20 cigarettes per day				
		Non smokers			.95 Confidence Intervals					.95 Confidence Intervals		
GENE/ RS NO.	GENOTYPE	NO.	NO.	Odds Ratio	Lower Limit	Upper Limit	p*	NO.	Odds Ratio	Lower Limit	Upper Limit	p*
DROSHA												
**6877842**	CC	0	1	Ref.				5	Ref.			
	CG	8	11	NA				30	NA			
	GG	3	13	NA				57	NA			
DICER1												
**3742330**	AA	3	8	Ref.				12	Ref.			
	AG	7	17	0.9107	0.1853	4.4763	0.6204 F	76	2.7143	0.6159	11.9625	0.1785
	GG	0	0	NA				0	NA			
**13078**	AA	0	2	Ref.				5	Ref.			
	AT	4	7	NA				35	NA			
	TT	7	18	NA				50	NA			
DGCR8												
**1640299**	GG	0	1	Ref.				5	Ref.			
	GT	4	14	NA				29	NA			
	TT	5	9	NA				46	NA			
**3757**	AA	0	0	Ref.				4	Ref.			
	AG	7	15	NA				67	NA			
	GG	3	8	NA				18	NA			
**417309**	AA	1	5	Ref.				7	Ref.			
	AG	3	7	0.4667	0.0369	5.9028	0.5109 F	22	1.0476	0.0934	11.755	0.6908 F
	GG	6	11	0.3667	0.0344	3.9079	0.3822 F	50	1.1905	0.1242	11.4082	0.6266 F
RAN												
**14035**	CC	6	12	Ref.				55	Ref.			
	CT	1	9	0.0556	0.0056	0.5468	0.0051 F	22	2.4	0.2729	21.1044	0.3782 F
	TT	1	1	0.5	0.0264	9.4579	0.5894 F	3	0.3273	0.0292	3.6621	0.3733 F
TARBP2												
**784567**	AA	1	1	Ref.				5	Ref.			
	AG	3	14	4.6667	0.2234	97.5022	0.3859 F	53	0.283	0.0246	3.2533	0.3415 F
	GG	6	13	2.1667	0.115	40.8129	0.5666 F	32	0.3019	0.0705	1.2918	0.0929 F
XPO5												
**11077**	GG	2	7	Ref.				17	Ref.			
	GT	6	13	0.619	0.0978	3.9188	0.4844 F	43	0.8431	0.1546	4.5968	0.6052 F
	TT	3	6	0.5714	0.0703	4.6446	0.5 F	27	1.0588	0.1601	7.0041	0.6522 F

*All p values are for Fisher Exact Probability Test due to low sample sizes.
